# WAXSiS: a web server for the calculation of SAXS/WAXS curves based on explicit-solvent molecular dynamics

**DOI:** 10.1093/nar/gkv309

**Published:** 2015-04-08

**Authors:** Christopher J. Knight, Jochen S. Hub

**Affiliations:** Institute for Microbiology and Genetics, Georg-August-University Göttingen, Justus-von-Liebig-Weg 11, 37077 Göttingen, Germany

## Abstract

Small- and wide-angle X-ray scattering (SWAXS) has evolved into a powerful tool to study biological macromolecules in solution. The interpretation of SWAXS curves requires their accurate predictions from structural models. Such predictions are complicated by scattering contributions from the hydration layer and by effects from thermal fluctuations. Here, we describe the new web server WAXSiS (WAXS in solvent) that computes SWAXS curves based on explicit-solvent all-atom molecular dynamics (MD) simulations (http://waxsis.uni-goettingen.de/). The MD simulations provide a realistic model for both the hydration layer and the excluded solvent, thereby avoiding any solvent-related fitting parameters, while naturally accounting for thermal fluctuations.

## INTRODUCTION

Small- and wide-angle X-ray scattering (SAXS/WAXS or SWAXS) has been gaining increased popularity as a structural probe for biomolecules in solution (1–4). Traditionally, SAXS has been used to detect global parameters of biomolecules, such as the radius of gyration, the multimeric state, or aggregation. Thanks to advances in light sources, detectors, and sample preparation, SWAXS experiments have become increasingly powerful, allowing one to track small-scale conformational transitions, and allowing accurate measurements at wider angles (5). In parallel to experimental progress, a number of software packages were developed for SWAXS analysis and modelling, paving the way for SWAXS to become a standard tool for biomolecular research.

The interpretation of SWAXS curves requires their accurate predictions from structural models (6). However, such predictions are not trivial for a number of reasons. SWAXS is a contrast method, so the scattering from the displaced solvent must be subtracted from the scattering of the solute. Furthermore, the hydration layer on biomolecules contributes to the scattering signal. The density of the hydration layer is typically larger as compared to bulk solvent, leading to an apparently increased radius of gyration of the solute (7). In addition, the hydration layer has internal structure, which may contribute the scattering signal at wide angles. Apart from such complications with the solvent, thermal fluctuations have an effect on the scattering signal, in particular at wider angles (8,9).

A number of methods have been developed to predict SWAXS curves from structural models, and some of those are available as web servers ((6) and references therein). Popular methods such as CRYSOL, FoXS, AXES, AquaSAXS and sastbx use multiple fitting parameters to match the predicted with the experimental SWAXS curve (10–14). As a common feature, they use a fitting parameter associated with the density of the hydration layer, whereas the choice of additional fitting parameters, associated with the displaced solvent or the buffer subtraction, differs between these methods. Fitting procedures may lead to a good match between the predicted and the calculated curve, but they reduce the amount of available information that can be extracted from the SWAXS curves, and may lead to overfitting (12). In addition, it is difficult to account for thermal fluctuations by such methods.

Explicit-solvent molecular dynamics (MD) simulations overcome some of these limitations at higher computational cost (7,9,15–18). In a recent article, we showed that MD simulations accurately reproduce the increase of the radius of gyration due to the hydration layer, suggesting that the simulations provide an accurate model of hydration (9). Thus, SWAXS curves computed from MD simulations do not require fitting of the hydration shell, thereby avoiding the modification of the radius of gyration by the fitting procedure. Likewise, if the scattering by the excluded solvent is computed by explicit water and not by dummy atoms (as done by implicit solvent methods), no scaling parameters are required for the excluded solvent (9,12,16–18). Moreover, MD simulations naturally account for thermal fluctuations. However, SWAXS predictions based on MD are so far not accessible to non-experts. We therefore present a new web server termed WAXSiS (WAXS in solvent) that computes SWAXS curves based on explicit-solvent MD simulations (http://waxsis.uni-goettingen.de/). Given the structure of a biomolecule, the server automatically runs an MD simulation and computes the SWAXS curve using the methods outlined in a recent article (9). If the user provides an experimental scattering curve *I*_exp_(*q*), the server fits the experimental to the calculated curve following *I*_fit_(*q*) = *fI*_exp_(*q*) + *c*. Thus, besides the arbitrary overall scale *f*, only one additional parameter *c* is fitted that aims to absorb some experimental uncertainty due to the buffer subtraction. In contrast to implicit solvent methods, neither the density of the hydration layer nor the density of the of the excluded solvent are fitted to the experiment.

Scattering intensities are typically recorded separately for the sample, *I*_sam_(*q*), and for the buffer, *I*_buf_(*q*). SWAXS intensities are reported as the difference in intensity between sample and buffer, yet two different buffer subtraction schemes are frequently used in the literature:

(1) \begin{eqnarray*} I(q) &=& I_\text{sam}(q) - I_\text{buf}(q), \end{eqnarray*}

(2) \begin{eqnarray*} I(q) &=& I_\text{sam}(q) - (1-v) I_\text{buf}(q). \end{eqnarray*}

Following the second scheme, the buffer intensity is reduced by the volume fraction *v* taken by the solute. Net intensities computed by the two subtraction schemes differ slightly (yet significantly) at small angles, and they differ highly at wide angles where the water scattering becomes dominant. Both subtraction schemes are supported by WAXSiS.

## WAXSIS METHOD

### SWAXS calculation

The WAXSiS server computes the SWAXS curves following the methods described in a recent article (9). Accordingly, WAXSiS runs an explicit-solvent MD simulation of the biomolecule, typically for 20–500 ps depending on the size of the biomolecule. During the simulation, position-restraining potentials (force constant: 1000 kJ/mol nm^2^) are applied to the backbone atoms of the biomolecule and on heavy atoms of ligands. That procedure ensures that the simulation samples conformations close to the initial structure, while allowing thermal fluctuations of side chains, water and counter ions. The scattering contribution from the excluded solvent is computed from an MD trajectory of a pure-water simulation system, which is stored on the WAXSiS server.

**Figure 1.**
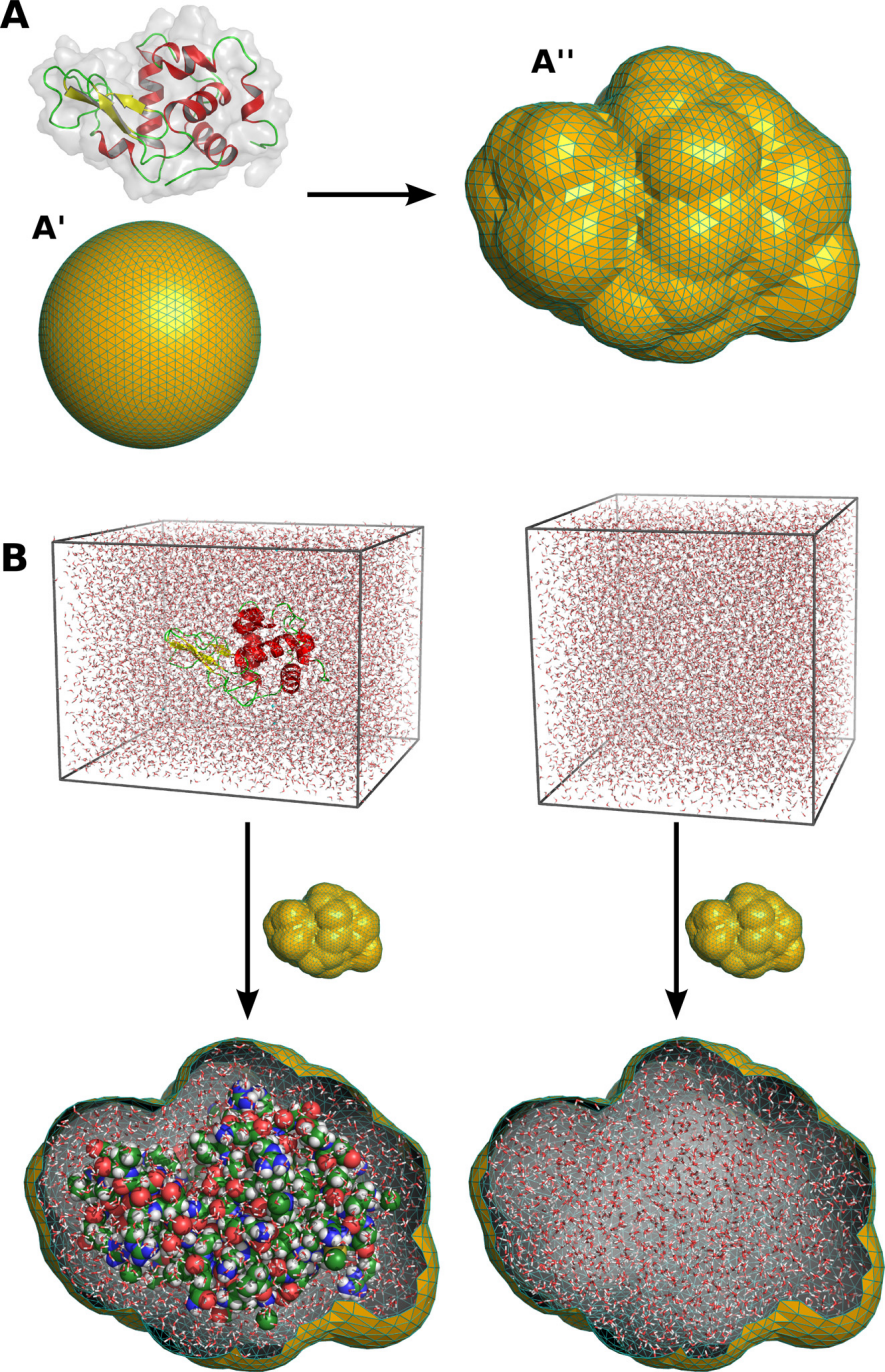
Construction of the hydration layer and excluded solvent using a spatial envelope. (**A**) The envelope is built from an icosphere (**A**′), which is obtained from a regular isosahedron by recursively subdividing is triangular faces into smaller triangles. Subsequently, the envelope is by default constructed at a distance of 7 Å from all solute atoms of all simulation frames (A''). (**B**) The hydration layer (left) and the excluded solvent (right) are obtained as the atoms inside of the envelope, overlayed with the solute or pure-solvent simulation system, respectively.

After the solute simulation has finished, a spatial envelope is constructed that encloses the solute at a preselected distance (7 Å by default). Our algorithm constructs the envelope from an icosphere, which is obtained from a regular icosahedron by recursively subdividing its triangluar faces into four smaller triangles. An icosphere after four such recursions is shown in Figure 1A. The envelope is then obtained by moving the vertices of the icosphere in a radial direction, until all vertices have a distance of at least *d* from all solute atoms in all simulation frames. Once constructed, the same envelope is applied throughout the remaining calculation. Molecular images in Figure 1 were rendered with PyMol (19).

The electron density of each simulation frame is decomposed into density inside and outside of the envelope:

(3) \begin{eqnarray*} A(\boldsymbol {r}) &=& A_{\rm i}(\boldsymbol {r}) + A_{\rm o}(\boldsymbol {r}), \end{eqnarray*}

(4) \begin{eqnarray*} B(\boldsymbol {r}) &=& B_{\rm i}(\boldsymbol {r}) + B_{\rm o}(\boldsymbol {r}). \end{eqnarray*}

$A(\boldsymbol {r})$ and $B(\boldsymbol {r})$ denote the electron density in the solute and pure-water simulation systems, respectively, and the subscripts i and o indicate density inside and outside of the envelope. An example for the atoms contributing to $A_{\rm i}(\boldsymbol {r})$ and $B_{\rm i}(\boldsymbol {r})$ is shown in Figure 1B. As shown previously (9,17), the net intensity is then given by

(5) \begin{eqnarray*} I(q) &=& \left\langle D(\boldsymbol {q}) \right\rangle _\Omega \end{eqnarray*}

(6) \begin{eqnarray*} D(\boldsymbol {q}) &:=& \big \langle | \tilde{A}_{\rm i}(\boldsymbol {q}) |^2 \big \rangle ^{(\omega )} - \big \langle | \tilde{B}_{\rm i}(\boldsymbol {q}) |^2 \big \rangle ^{(\omega )} \nonumber \\ && +2\rm{Re}\left[ -\big \langle \tilde{B}_{\rm i}^\ast (\boldsymbol {q}) \big \rangle ^{(\omega )} \big \langle \tilde{A}_{\rm i}(\boldsymbol {q}) - \tilde{B}_{\rm i}(\boldsymbol {q}) \big \rangle ^{(\omega )}\right], \end{eqnarray*}

Here, $\tilde{A}_{\rm i}(\boldsymbol {q})$ and $\tilde{B}_{\rm i}(\boldsymbol {q})$ are the Fourier transforms of the densities $A_{\rm i}(\boldsymbol {r})$ and $B_{\rm i}(\boldsymbol {r})$, respectively. 〈·?^(ω)^ denotes the average over the simulation frames, while the superscript highlights that this average is taken at a fixed solute orientation ω. The asterisk indicates complex conjugate. $\left\langle \cdot \right\rangle _\Omega$ denotes the orientational average, taking account of the fact that the solutes are randomly oriented in solution. Hence, Equations (5) and (6) show that only the densities *inside* of the envelope contribute, justifying why *I*(*q*) can be computed from a finite simulation system. The three terms in Equation (6) have an intuitive interpretation; the first term corresponds to the scattering of the solute and its hydration layer, and the second to the scattering of the excluded solvent. The third term corresponds to correlations between bulk water and the density contrast inside the envelope (9). If the buffer subtraction scheme of Equation (2) is applied, *I*(*q*) is corrected following the procedure in (18).

Given the coordinates of atoms within the envelope, the scattering amplitude for an individual simulation frame is written as

(7) \begin{equation*} \tilde{A}_{\rm i}(\boldsymbol {q}) = \sum _{j=1}^{N_{\rm A}} f_j(q)\,\rm{e}^{-i\boldsymbol {q}\cdot \boldsymbol {r}_j}, \end{equation*}

where *N*_A_ is the number of atoms within the envelope, *f*_*j*_(*q*) are the atomic form factors, and $\boldsymbol {r}_j$ is the coordinate of atom *j*. The *f*_*j*_(*q*) are computed following $f_j(q) = \sum _{k=1}^4 \,a_k\,\exp [-b_k(q/4\pi )^2] + c$, where *a*_*k*_, *b*_*k*_, *c* are the Cromer–Mann parameters (20), which are published in tables (21). The analogous relation is applied to compute $\tilde{B}_{\rm i}(\boldsymbol {q})$ using the atoms inside the envelope of the pure-water system (see Figure 1B). In order to account for electron-withdrawing effects in water molecules, we apply the correction proposed by Sorenson *et al*. to the form factors of water atoms (22).

The orientational average is evaluated numerically. For each absolute value of the scattering angle *q*, a set of vectors $\boldsymbol {q}_j$ (*j* = 1, …, *J*_*q*_) is distributed uniformly on the surface of a sphere with radius *q*. The vectors $\boldsymbol {q}_j$ are distributed following the spiral method, as done previously (9,17). In our previous article we tested the convergence of *I*(*q*) with *J*_*q*_, using various *q* and different solutes. In line with theoretical considerations (23), we observed that *J*_*q*_ should be taken proportional to (*qD*)^2^ to achieve a preselected convergence, where *D* denotes the maximum diameter of the solute. In WAXSiS, we therefore use *J*_*q*_ = Max{100, 0.2 · (*qD*)^2^}, leading to small uncertainties over the entire *q*-range.

The computed SWAXS curves are at small angles very sensitive to small alternations in the solvent density. Because the density of the experimental buffer might deviate from the density of the explicit solvent model, we correct the solvent density following the method described previously (9). In short, we add a small uniform electron density to the water density, both in the bulk water and in the solute simulation system, to match the bulk density with a preselected buffer density. By default, we use a buffer density of 334 e nm^−3^, corresponding to pure water, but the WAXSiS user may specify a different buffer density. It is important to note that we do *not* fit the solvent density to match the computed with the experimental intensity.

### Fitting procedure

If the user uploads an experimental SWAXS curve *I*_exp_, it is fitted to the predicted curve (and not vice versa), because the predicted curve does not contain any free parameters. WAXSiS fits *I*_exp_ by minimizing two different metrics,

(8) \begin{eqnarray*} \chi ^2(f,c) &=& N^{-1}\sum _{i=1}^N \left[ \frac{I_\text{calc}(q_{\rm i}) - (f I_\text{exp}(q_{\rm i}) +c )}{\sigma _\text{exp}(q_{\rm i})} \right]^2 \end{eqnarray*}

(9) \begin{eqnarray*} &&\chi _\text{log}^2(f,c) \nonumber \\ &=& N^{-1}\sum _{i=1}^N [ \log I_\text{calc}(q_{\rm i}) - \log (f I_\text{exp}(q_{\rm i}) +c )]^2, \end{eqnarray*}

where *N* denotes the number of *q*-points, σ_exp_(*q*_*i*_) are the experimental errors, and *f* and *c* the fitted parameters. Thus, besides the overall scale *f*, only a constant offset *c* is fitted to absorb some uncertainty in the buffer subtraction. The first metric (Equation 8) is weighted by the experimental errors and is widely used in SWAXS analysis, but it may impose spuriously high weights to small angles. The second metric (Equation 9) imposes more uniform weights over small and wide angles, and we found it to be particularly suitbable to interpret wide-angle data. The fitted curve and χ-value for each metric are reported by WAXSiS.

### MD simulation setup

The MD simulations are conducted by the YASARA Dynamics software (YASARA Biosciences, Vienna, Austria). YASARA is capable of setting up MD simulations automatically from PDB structures, thereby adding missing hydrogen and heavy atoms, fixing steric problems, and correcting for other frequent imperfections in PDB files. Protein and nucleic acids are described by the AMBER03 force field (24), and water parameters are taken from the TIP3P model (25). Force field parameters for modified side chains or ligands are derived by YASARA's AutoSMILES method. Should the structure contain exotic metal ions for which no force field parameters are available, WAXSiS replaces them with Fe^2 +^ before running YASARA. For the calculation of SWAXS curves, however, the form factors of the original exotic ions are applied. Electrostatic interactions are computed using the particle-mesh Ewald method, and the dispersive interactions are described by a Lennard-Jones potential with a cutoff at 9 Å. Temperature is controlled at 298.15 K and the pressure at 1 bar using YASARA's default temperature and pressure control settings. Simulation frames are written every 0.5 ps to ensure that the solvent configurations are reasonably uncorrelated. Based on the convergence assessment in previous work (9), we compute the total number of generated MD frames as $N_\text{fr} = 2\times 10^5 / N_A^{0.77}$. Note that *N*_fr_ can also be controlled by the user option “Convergence” (see below). Before collecting simulation frames for the SWAXS calculation, the simulation system is equilibrated for 5% of the total simulation time (but not shorter than 3 ps).

## WAXSIS WEB SERVER

**Figure 2.**
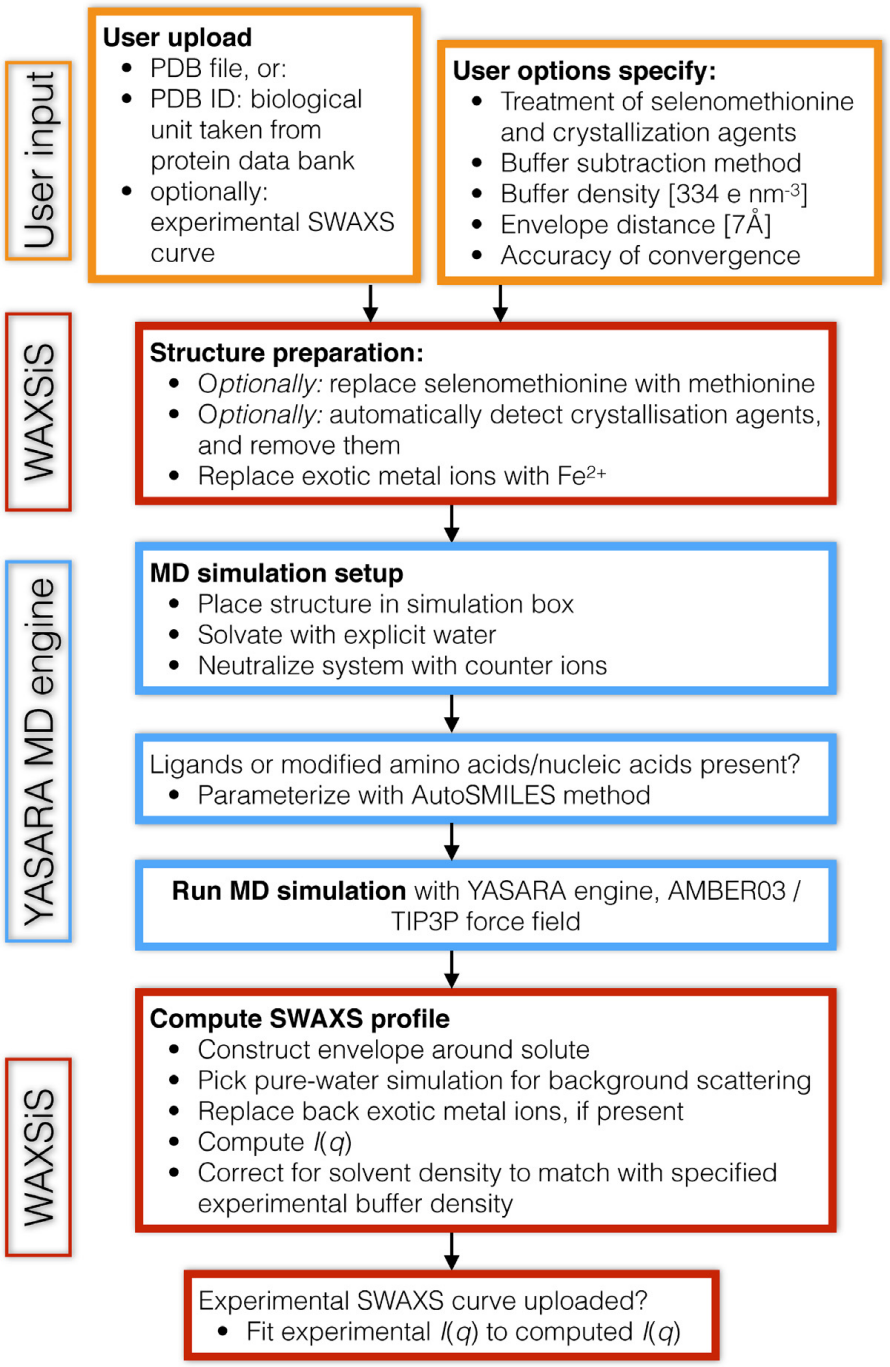
Workflow of a WAXSiS job. Orange boxes indicate user input, blue boxes indicates workflow conducted by YASARA and red boxes other WAXSiS workflow.

The WAXSiS server has a flexible user frontend that can be accessed from a computer, tablet, or smartphone. The web site provides many details on the method of SWAXS calculation in the About section, as well as a list of frequently asked questions (FAQs) in the Help section. The workflow of a WAXSiS job is illustrated in Figure 2.

### Specifying the initial structure

The user may either upload structure of a biomolecule or specify a protein data bank (PDB) code. In the latter case, WAXSiS picks the biological assembly of that PDB code from the PDB. If multiple biological assemblies are stored for this PDB entry, the assembly may be specified. If no biological assembly is present, the user is notified and the crystallographic structure is taken instead. Finally, if the PDB code corresponds to an NMR ensemble, the first structure from the ensemble is taken, leading to a note in the job results.

Many PDB structures contain crystallization agents, or methionine residues might have been replaced with selenomethionine to aid solving the structure. Because such modifications are typically not present in the SWAXS sample, WAXSiS by default replaces selenomethionine with methionine side chains. In addition, WAXSiS tries to identify crystallization agents by default and removes them. Because PDB files do not contain reliable information on whether a molecule is a crystallization agent or a biologically relevant ligand, WAXSiS tries to detect crystallization agents using a combination of ligand size, number of atomic contacts, and residue name. Specifically, molecules are considered biologically relevant if one of the following criteria matches: (i) they form a covalent bond the biomolecule; (ii) they form more than 2.5 contacts per heavy atom to the biomolecule (contact cutoff 3.5 Å); (iii) they contain >20 heavy atoms; (iv) the residue name is HEM, indicating a heme ligand. Molecules with residue names GOL, BNG, BOG, PG, PE, PGE typically indicate glycerol, B-nonylglucoside, B-octylglucoside, and polyethylene glycol, which are always considered as crystallization agents. These criteria worked reliably for many test cases. Both the handling of crystallization agents and selenomethionine can be specified by the user. If in doubt, we recommend that the user removes unwanted molecules manually, uploads the structure, and specifies WAXSiS to 'Keep both ligands and crystallization agents' in the user options.

### Input options

The following basic options can be specified by the user. Default values are printed in italic font:

- Email address (optional): if an email address is entered, the user will be notified when the job has finished.
- Ligand handling: (i) *keep ligands, try to remove crystallization agents*, (ii) keep ligands and crystallization agents, (iii) remove all.
- Buffer subtraction method: (i) *buffer scattering reduced by solute volume* (Equation 2), (ii) total buffer scattering subtracted (Equation 1).
- Maximum scattering vector *q*, defined as *q* = 4πλ^−1^sin θ, where 2θ is the scattering angle (default: 1 Å^−1^)
- Optional upload of an experimental SWAXS curve. The *q* units (Å^−1^ or nm^−1^) and the scattering convention (*q* or *s* = 2λ^−1^sin θ) adopted for the experimental data can be specified.

The following advanced options can be specified:

- Output *q* units (Å^−1^ or nm)
- Electron density of the buffer (default: 334 e nm^−3^)
- Replace selenomethionine with methionine (*yes*/no)
- Distance of envelope from the solute (default: 7 Å)
- Accuracy of convergence (quick/*normal*/thorough). Selecting 'quick' or 'thorough' causes WAXSiS to run the MD simulation four times shorter or five times longer as compared to 'normal' convergence, respectively. “Thorough” jobs run at lower priority in the queue.
- Generate a new random seed for initial velocities. Choosing yes/no leads to slightly varying or reproducible results, respectively.

### Output

**Figure 3.**
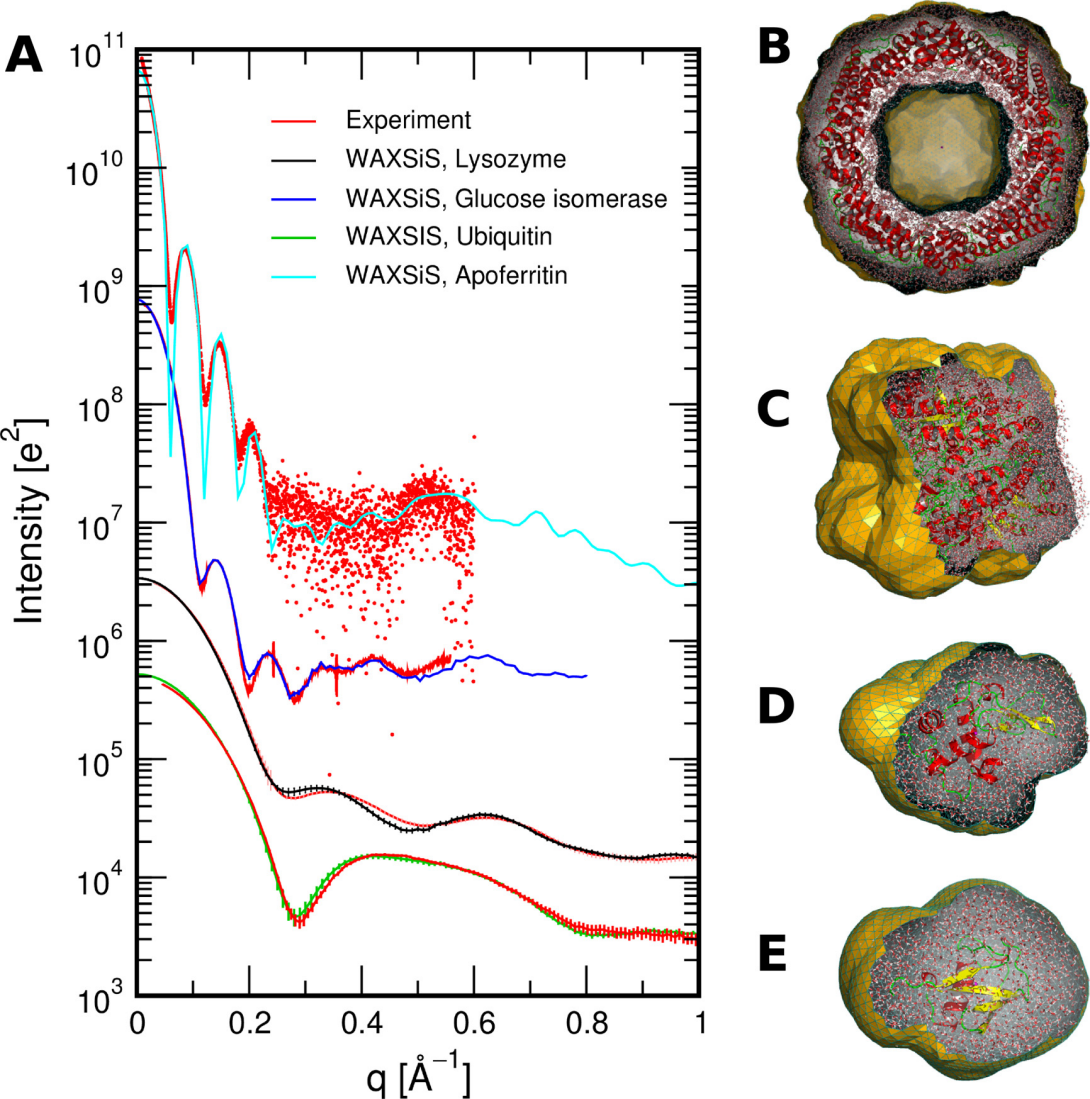
(**A**) SWAXS curves computed by WAXSiS for a number of proteins, as listed in the legend, computed from PDB structures 1ier, 1mnz, 1lys, and 1d3z. Red curves show fitted experimental data, taken from the Small Angle Scattering Biological Data Bank (26) (SASDA82 and SASDAK6) and from (12). (B–E) Molecular representations of solute and hydration layer. The envelope (orange) is clipped for visualization. (**B**) Apoferritin, together with its envelope with a hole at the center, (**C**) glucose isomerase, (**D**) lysozyme, (**E**) ubiquitin.

WAXSiS jobs generate graphical, data file, and structure file output. The results are summarized on a web page with a randomized URL to ensure user privacy. The web page shows the SWAXS curve, together with the fitted experimental curve (if present), as well as a Guinier fit. In addition, molecular representations of solute, hydration layer and excluded solvent are shown. The web page also presents a link to a gzipped tar ball that contains the following detailed results:

- Data and PDF files of the calculated SWAXS curve and Guinier fit, as well as the fitted experimental curve (if provided). Figure 3A shows a few examples of SWAXS curves computed by WAXSiS, together with fitted experimental curves. Experimental data shown in Figure 3A was taken from the Small Angle Scattering Biological Data Bank (26) (SASDA82 and SASDAK6) and from (12).
- A log file reporting details on the job options, ligand handling, MD simulation, SWAXS calculation, buffer subtraction scheme, fitting results and Guinier fit.
- A PDB file complete.pdb of the initial frame of the MD simulation system, containing the solute (including hydrogen atoms), water and counter ions. In addition, PDB files of the solute and hydration layer as well as of the excluded solvent are included, taken from the first frame of the MD trajectory after equilibration.
- A dummy topology file in Gromacs format. This file lists which element and hence, atomic form factor, was assigned to each atom. The atom numbers correspond to the atoms in complete.pdb. The user may consult this file to check if WAXSiS assigned the correct elements to uncommon atoms, such as exotic metal ions.

### SWAXS curves from uploaded MD trajectories

As an additional feature, WAXSiS computes SWAXS curves based on explicit-solvent MD simulations conducted by the user. The user uploads the complete simulation system as a PDB file, the simulation trajectory (in Gromacs format), as well as an index file (in Gromacs format) defining the solute and the solvent. Details are provided in the Help section of WAXSiS. It is important to note that the simulation system should contain a rather large amount of water to ensure that the constructed envelope fits into the simulation cell.

## CONCLUSION

We described a new web server called WAXSiS for the calculation of SAXS/WAXS curves of biomolcules. Unlike other web servers, WAXSiS is based on explicit-solvent molecular dynamics simulations. Thus, WAXSiS uses a highly accurate model for the hydration layer of the biomolcules and for the excluded solvent. In addition, the simulations account for thermal fluctuations of water, side chains, and counter ions. WAXSiS does not require any fitting parameters associated with the hydration layer or excluded solvent, rendering the calculations highly predictive.
